# Should Prophylactic Anticoagulation Be Considered with Large Uterine Leiomyoma? A Case Series and Literature Review

**DOI:** 10.1155/2016/9803250

**Published:** 2016-11-03

**Authors:** Mohamed A. Satti, Carmen Paredes Saenz, Rubin Raju, Sierra Cuthpert, Abed Kanzy, Sina Abhari, John Hebert III, Frederico G. Rocha

**Affiliations:** ^1^Department of Obstetrics and Gynecology, Hurley Medical Center/Michigan State University College of Human Medicine, Flint, MI 48503, USA; ^2^Department of Internal Medicine, Hurley Medical Center/Michigan State University College of Human Medicine, Flint, MI 48503, USA

## Abstract

*Introduction*. Uterine leiomyomas, also called uterine fibroids or myomas, are the most common pelvic tumors in women. They are very rarely the cause of acute complications. However, when complications occur they cause significant morbidity and mortality. Thromboembolic disease has been described as a rare complication of uterine leiomyomas. DVT is a serious illness, sometimes causing death due to acute PE.* Cases*. We report a case series of 3 patients with thromboembolic disease associated with uterine leiomyoma at Hurley Medical Center, Flint, Michigan, during 2015 and conduct a literature review on the topic. A literature search was conducted using Medline, PubMed, and PMC databases from 1966 to 2015.* Conclusion*. The uterine leiomyoma is a very rare cause of PE and only few cases have been reported. DVT secondary to uterine leiomyoma should be considered in a female presenting with abdominal mass and pelvic pressure, if there is no clear common cause for her symptoms. Thromboembolic disease secondary to large uterine leiomyoma should be treated with acute stabilization and then hysterectomy. Prophylactic anticoagulation would be beneficial for lowering the risk of VTE in patients with large uterine leiomyoma.

## 1. Introduction

Uterine leiomyomas, also called uterine fibroids or myomas, are the most common pelvic tumors in women [1]. Myomas arise as monoclonal tumors from smooth muscle cells of the myometrium. Symptoms related to uterine leiomyomas can be classified as follows: heavy or prolonged uterine bleeding, reproductive dysfunction, and pelvic pressure and pain [2].

Although uterine leiomyomas constitute the most common tumor in women of reproductive age, they are very rarely the cause of acute complications. However, when complications occur they cause significant morbidity and mortality. The acute complications include thromboembolism, torsion of subserosal pedunculated leiomyomata, urinary retention and renal failure, acute pain caused by red degeneration during pregnancy, vaginal or intraperitoneal hemorrhage, mesenteric vein thrombosis, and intestinal gangrene.

Thromboembolic disease has been described as a rare complication of uterine leiomyomas. There are only few reports [3–25] describing this association in patients with no other risk factors for venous thromboembolism (VTE).

Deep venous thrombosis (DVT) is a serious illness, sometimes causing death due to acute pulmonary embolism (PE).

## 2. Aim

The aim of this study is to report a case series of 3 patients with thromboembolic disease associated with uterine leiomyoma at Hurley Medical Center, Flint, Michigan, during 2015 and to conduct a literature review on the topic.

## 3. Case 1

A 47-year-old woman who was recently discharged from our institution after she was treated for pneumonia and anemia presented with complaints of left leg swelling and pain for 2 days. She had no history of recent trauma or recent travel. She was not on any oral contraceptive pills. She denied any history of prior thromboembolic events and there was no family history of DVT or cancer. Review of systems was positive for pelvic pressure, urinary urgency, and menorrhagia. Physical examination was remarkable for enlarged firm abdominal mass and left thigh and leg swelling with tenderness.

Laboratory findings were unremarkable except for hemoglobin of 6.9 mg/dL. Left lower extremity venous Doppler showed acute DVT in the left common femoral vein, femoral, profunda femoral veins, and popliteal and tibial peroneal veins. A computed tomography (CT) scan of the chest confirmed a left lower lobe lung PTE. An abdominal CT scan showed an enlarged uterus measuring 22 × 8.8 × 14 cm with numerous fibroids, the largest of which measured approximately 7 cm. The patient was treated acutely with unfractionated heparin and warfarin. Inferior vena cava (IVC) filter was placed to decrease the risk of perioperative pulmonary embolism. She was then evaluated by gynecology for treatment of the enlarged uterus. Patient was discharged home, but she never showed up for her follow-up appointment.

## 4. Case 2

A 60-year-old woman, para 0, with known history of large uterine fibroid presented to the emergency department (ED) in our institution with complaints of dyspnea and left lower back pain, which appeared to be pleuritic in nature for 5 days. Patient denied history of prior DVT, PE, or recent immobilization. On physical examination, she was anxious, tachypneic, and tachycardic with BMI 17.7. Lung examination revealed bilateral rhonchi and slightly decreased breath sounds at the left posterolateral lung field. Other findings included a nontender abdomen with a 30-week size uterus. There was no edema in her lower extremities.

**Figure 1:**
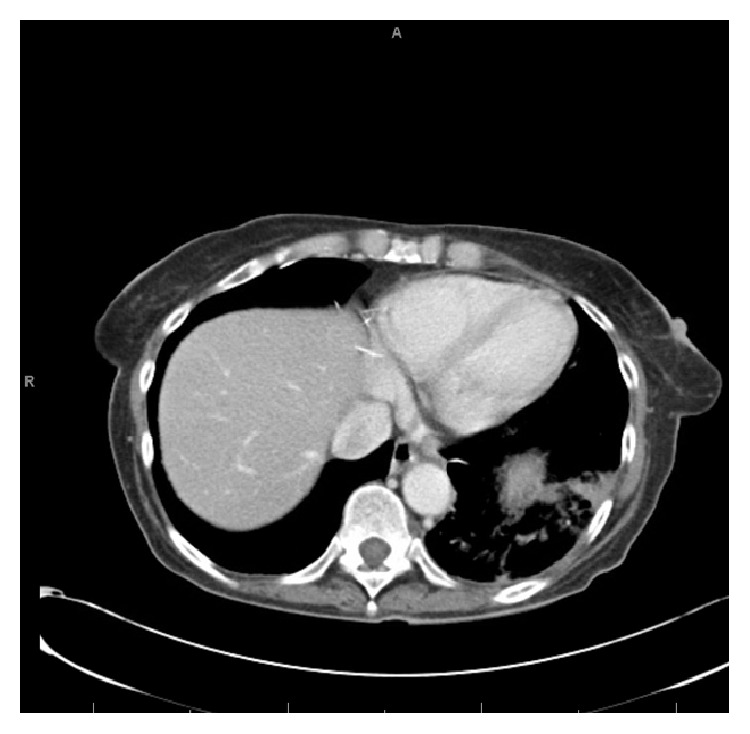
Chest CT scan of case 2 showing pulmonary embolism.

**Figure 2:**
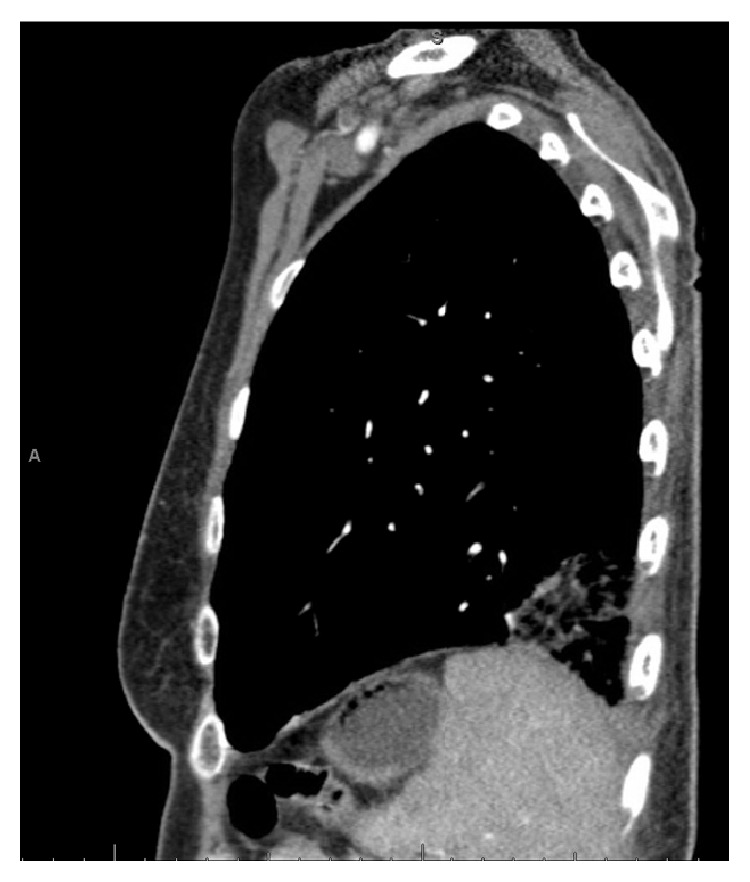
Chest CT scan of case 2 showing pulmonary embolism.

**Figure 3:**
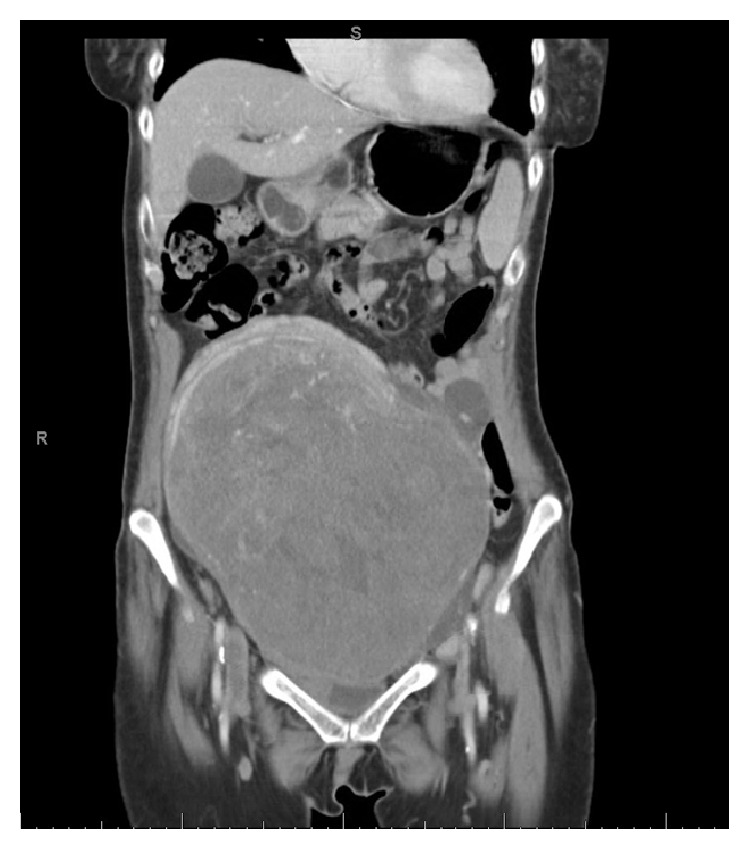
Abdomen-pelvis CT scan of case 2 showing large uterine leiomyoma.

**Figure 4:**
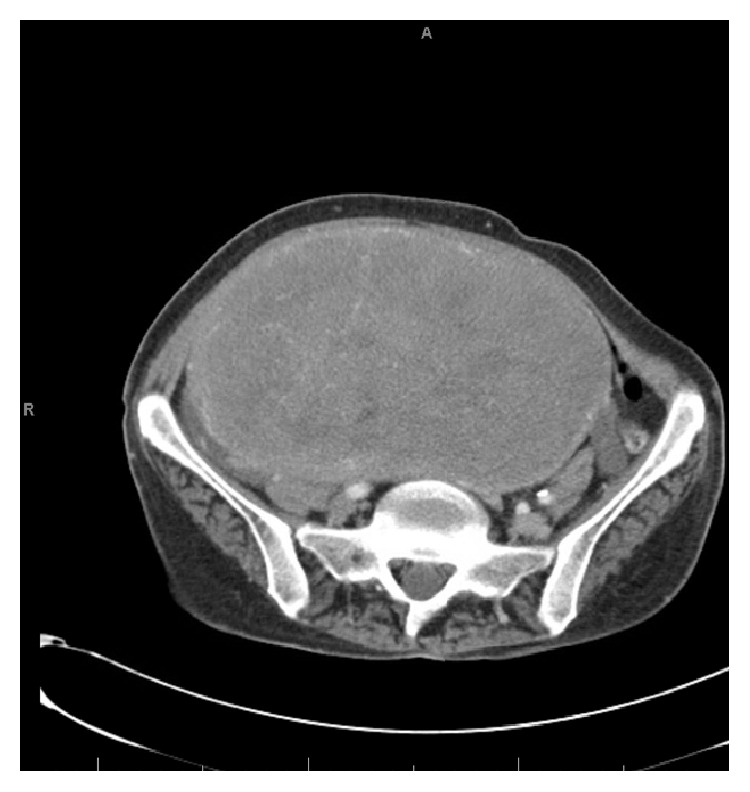
Abdomen-pelvis CT scan of case 2 showing large uterine leiomyoma.

A chest radiograph was not significantly abnormal. A vascular ultrasound investigation revealed acute DVT involving bilateral lower extremity veins. Thereafter, a chest spiral CT scan confirmed the presence of a left lower lobe lung PE with possible beginnings of a pulmonary infarct in the left lower lobe (Figures 1 and 2). Patient underwent an abdominal CT scan, which confirmed a huge uterine mass measuring 23.5 × 14 × 21.2 cm (Figures 3 and 4).

The patient was admitted to the Intensive Care Unit (ICU) and was treated with unfractionated heparin and warfarin. Since the uterine myoma was considered one of the major causes of DVT and PE, total hysterectomy was planned. Accordingly, to prevent recurrence and extension of PE during gynecologic procedure a Günther-Tulip vena cava filter was preoperatively inserted via the jugular route into the inferior vena cava.

Total abdominal hysterectomy and bilateral salpingoophorectomy were carried out under a general anesthesia. The histopathologic diagnosis was a large myomatous uterus that weighted 3010 grams with normal tubes and atrophic change with corpora albicantia in both ovaries.

## 5. Case 3

A 41-year-old nonsmoking woman, gravida 1, para 1, was presented to the ED in our institution with vaginal bleeding, dizziness, and shortness of breath. The past medical history was significant for arterial hypertension, type 2 diabetes mellitus, peripheral artery disease, and uterine fibroids. Patient reported menorrhagia that had been worsening over the past year, with resultant severe iron-deficiency anemia requiring multiple blood transfusions. There was no personal or family history of coagulopathies.

Physical examination revealed generalized pallor and a nontender abdomen with a 28-week size uterus and BMI 35.9. Laboratory findings showed hemoglobin level of 3.3 g/dL, hematocrit of 13.8, mean corpuscular volume 65, prothrombin time 15.5, and thromboplastin time 25.4. Blood products transfusion was ordered and patient received a total of 8 units of red blood cells and 2 units of fresh frozen plasma. An abdominal ultrasound showed a significantly enlarged uterus 18 × 13 × 14.9 cm extending above the level of umbilicus, containing several intrauterine masses, most consistent with fibroids.

Gynecology team was consulted for management of uterine fibroids; accordingly cervical cytology and endometrial biopsy were done and patient was started on oral provera, then her vaginal bleeding subsided, and she was discharged home in stable condition. Cervical cytology was negative for intraepithelial lesion or malignancy and endometrial biopsy showed an anovulatory endometrium. The patient was scheduled for a total abdominal hysterectomy with bilateral salpingectomy one week later but unfortunately suffered a massive PE and expired before her scheduled surgery.

## 6. Literature Review

A literature search was conducted using Medline, PubMed, and PubMed Central (PMC) databases from 1966 to 2015. The following terms were used: “thrombosis”, “thromboembolic disease”, “pulmonary embolism”, “uterine myoma”, “uterine fibroid”, and “leiomyoma”. The reference lists of the selected articles were manually reviewed for additional articles. Nineteen articles were included with a total of 25 cases reporting the association between uterine leiomyoma and thromboembolic disease. In almost all cases, there were no other risk factors for venous thromboembolism.  Table 1 summarizes the literature review.

## 7. Discussion

Uterine leiomyomas are the most common pelvic tumors in women, occurring in 20–30% of women over 30 years of age. Approximately 50% of these women remain asymptomatic, while the other 50% present a diversity of symptoms. Most experts consider 10-centimeter diameter as large uterine leiomyoma [26]. Acute complications are rare; however when they do occur they can produce significant morbidity and affect a woman's quality of life [21, 27, 28]. Despite the frequency of uterine leiomyomas, its association with thromboembolic disease is uncommon and has been reported only in few case reports (Table 1). Venous thromboembolic disease includes deep venous thrombosis and pulmonary embolism. Both conditions are associated with significant morbidity and mortality. The average incidence of DVT is about 0.5 per 1000 person-years [29] and increases noticeably with age [30]. At least 100 000 annual deaths are caused by pulmonary embolism in the United States [31]. Virchow's triad explains the pathogenesis of thromboembolic disease. This theory proposes that VTE occurs as a result of (1) hypercoagulability, either systemic or local, (2) stasis of the venous blood, and (3) vessel wall injury, specifically in the endothelium. The proposed mechanism by which a large uterine leiomyoma may be associated with thromboembolic events is thought to be extrinsic mechanical compression of surrounding structures, including the pelvic venous system, leading to stasis and subsequent thrombosis [21, 27].

There are many risk factors for VTE. In general we can classify them to hereditary and acquired factors. The most common hereditary causes are inherited thrombophilia such as Factor V Leiden and prothrombin gene mutations. The major acquired factors include malignancy, recent major surgery, prior thrombotic event, pregnancy, oral contraceptives, hormone replacement therapy, immobilization, antiphospholipid antibody syndrome, and myeloproliferative disorders [32]. Blood transfusion might have an effect on thromboembolic disease, for example; red blood cell (RBC) and platelet transfusions are associated with an increased risk of venous and arterial thrombotic events and mortality only in hospitalized cancer patients and postoperative procedures [33, 34]. Each unit of fresh frozen plasma (FFP) increased VTE risk by 25% in patients who required less than 4 units of RBC. FFP administration conferred no increased risk of VTE in patients who required 4 units or greater RBC [35].

We could not find any set guidelines for managing patients with uterine fibroids and concomitant DVT. Brewer et al. recommend a multifaceted approach once the diagnosis of concomitant extensive DVT has been made, involving immediate anticoagulation for infra-inguinal DVT or thrombolysis/thrombectomy for more extensive DVT. They recommended fibroid removal prior to more definitive thrombosis management to reduce DVT recurrence [36]. Fletcher et al. highlighted the controversy of the use of anticoagulation in patients with menorrhagia due to the fear of worsening vaginal bleeding [37]. They also reviewed the need for reversal of anticoagulation prior to surgery to prevent primary hemorrhage and the use of mechanical compression stockings or low molecular weight heparin (LMWH) during surgery to avoid complications from venous stasis [37]. Pakiz and But report a case of large uterine fibroids with extensive DVT, managed with immediate anticoagulation followed by uterine artery embolization (UAE) to decrease uterine size allowing for resolution of the DVT and subsequent total abdominal hysterectomy with a successful outcome. Pakiz and But also describe the use of temporary retrievable filters but caution against the failure of retrieval of the filters in up to 15% necessitating lifelong anticoagulation [38].

Case 1 and case 2 were diagnosed with concomitant PE and DVT. As described above the presence of DVT secondary to the compression effect of large uterine fibroids likely preceded the PE in these patients. Duplex scan was used to diagnose DVT in case 1 and case 2. Case 3 did not have a duplex scan and presented with a massive PE and subsequently died. This would suggest benefit from routine screening using duplex scans for asymptomatic DVTs in patients with large uterine fibroids. Knowledge of an existing DVT would permit immediate anticoagulation therapy, thrombolytic therapy, and/or temporary IVC filter placement. This might prevent the occurrence of pulmonary embolisms in patients with large uterine fibroids. If the duplex scan does not show a DVT, a prophylactic anticoagulation might be considered till the patient gets a definite treatment. Of note, the American College of Chest Physicians Consensus guidelines for surgical patients does not recommend any type of anticoagulation for large uterine leiomyoma [39].

## 8. Conclusion

The uterine leiomyoma is a very rare cause of PE and only few cases have been reported. DVT secondary to uterine leiomyoma should be considered in a female presenting with abdominal mass and pelvic pressure, if there is no clear common cause for her symptoms. Thromboembolic disease secondary to large uterine leiomyoma should be treated with acute stabilization and then hysterectomy. To date we cannot suggest universal screening for VTE for all patients with large uterine leiomyoma; however compression ultrasound should be considered on cases where patients have additional risk factor for VTE. Prophylactic anticoagulation would be beneficial for lowering the risk of VTE in patients with large uterine leiomyoma and negative screening test till they are scheduled for surgery, unless they have vaginal bleeding.

## Figures and Tables

**Table 1:** All case reports of thromboembolic disease and uterine leiomyoma according to our literature review.

Reference	Year	Number of cases	Age	Diagnosis	Etiology	Treatment	Outcome
Ogawa et al.	1992	1	49 y.o	Lower extremity DVT + PE	Compression of the left external iliac vein	Pulmonary embolectomy + hysterectomy	Survived
Dekel et al.	1998	1	45 y.o	Lower extremity DVT	Compression of pelvic veins	Anticoagulation + IVC filter + hysterectomy	Survived
Chong et al.	1998	2	43 y.o	Lower extremity DVT	Compression of the IVC	IVC filter + hysterectomy	Survived
			49 y.o	Bilateral lower extremity DVT	No compression seen in images	Anticoagulation + hysterectomy	Survived
Nishikawa and Ideishi	2000	1	51 y.o	Lower extremity DVT + PE	Compression of pelvic vein	Anticoagulation + IVC filter + hysterectomy	Survived
Stanko et al.	2001	1	49 y.o	Lower extremity DVT	Compression of the left iliofemoral vein	Anticoagulation + IVC filter + hysterectomy	Survived
Phupong et al.	2001	1	42 y.o	Lower extremity DVT	Compression of the pelvic veins	Anticoagulation + hysterectomy	Survived
Tanaka et al.	2002	2	46 y.o	Lower extremity DVT + PE	Compression of pelvic veins	Anticoagulation + IVC filter + hysterectomy	Survived
			45 y.o	Lower extremity DVT	Compression of pelvic veins	Thrombolysis + anticoagulation + IVC filter + hysterectomy	Survived
Srivatsa et al.	2005	1	35 y.o	Bilateral internal iliac thrombi	Compression of the common iliac and inferior vena cava	Anticoagulants	Survived
Falcone and Serra	2005	1	39 y.o	Massive PE	Pelvic vein compression	Thrombolysis + anticoagulation + hysterectomy	Survived
Hawes et al.	2006	1	35 y.o	Lower extremity DVT	Compression of the distal inferior vena cava	Anticoagulants + hysterectomy	Survived
Bonito and Gulemi	2007	1	49 y.o	Lower extremity DVT + PE	Compression of pelvic veins	IVC filter + hysterectomy	Survived
Bekhit et al.	2007	1	55 y.o	Bilateral lower extremity DVT	Compression of pelvic veins + Tamoxifen use	IVC filter + hysterectomy	Survived
Khilanani and Dandolu	2007	1	44 y.o	Lower extremity DVT	Compression of inferior vena cava	IVC filter + hysterectomy	Survived
Asciutto and Mumme	2008	1	42 y.o	Lower extremity DVT	Compression of left iliac vein	Thrombectomy + hysterectomy	Survived
Kutsukata et al.	2009	1	42 y.o	Lower extremity DVT	Compression of inferior vena cava	Anticoagulation + thrombectomy + IVC filter + hysterectomy	Survived
Chandra et al.	2010	1	47 y.o	Lower extremity DVT	Compression of pelvic veins	Anticoagulation + hysterectomy	Survived
Huffman-Dracht and Coates	2010	1	37 y.o	Lower extremity DVT + PE	Compression of pelvic veins	IVC filter + anticoagulation	Loss to follow-up
Rosenfeld and Byard	2012	1	44 y.o	Lower extremity DVT + PE	Compression of pelvic veins	N/A	Died
Kurakazu et al.	2012	1	40 y.o	PE	Compression of iliac veins	Thrombolysis + hysterectomy	Survived
Srettabunjong	2013	1	46 y.o	Bilateral lower extremity DVT + massive PE	Compression of pelvic veins	N/A	Died
Toru et al.	2013	1	42 y.o	Lower extremity DVT	Compression at the bifurcation of the inferior cava	Anticoagulation	Loss to follow-up
Khademvatani et al.	2014	1	42 y.o	Lower extremity DVT + PE	Compression of common iliac veins	Thrombolysis + myomectomy	Survived
Fernandes et al.	2014	1	29 y.o	Lower extremity DVT + PE	Compression of iliac veins	Anticoagulation + hysterectomy	Survived
